# New Experiments on Mercurial Salts

**Published:** 1803-01-01

**Authors:** 


*New Experiments on Mercurial Salts, by* Cit. Fourcroy; *extracted from the Transactions of the Physical Class of the National Institute for the last three Months, and communicated by our Correspondent at Paris.*

THE Author had been already occupied in inquiries into the nature of these salts; he had already established three kinds of sulphat of mercury; the first a neutral salt, the second contained an excess of acid, and the third an excess of oxyd. These different salts are obtained by boiling one part of mercury with one and a half of sulphuric acid. If the operation be stopped, when the mercury is changed into a white mass, but not dried, you have the sulphat acid of mercury. If this sulphat acid be washed with a little water, until the water no longer changes vegetable blues, you have the neutral sulphat. The sulphat with excess of oxyd or turbith mineral, is prepared by heating one or other of the preceding salts, particularly the last, until the mercury has seized on part of the oxygen of the sulphuric acid, and until the sulphurous acid is disengaged. The neutral salt is soluble in 500 parts of cold water; it is precipitated by fixed alkalis of a grey colour.

It is not decomposed by nitric acid, and forms a mild, muriat with muriatic acid. It is composed of

| | |
|---|---|
| Acid | 12 |
| Mercury | 75 |
| Oxygen | 8 |
| Water | 5 |
| | 100 |

The salt, with excess of acid, is more soluble than the former, according to the excess of acid; it is precipitated yellow by fixed alkali, giving out much caloric, and is not decomposed by nitric acid; it differs from the preceding in its component parts only in the greater quantity of acid, and this last varies much. The salt with excess of oxyd or turbith mineral is yellow; it is not soluble in less than 2000 parts of water, is precipitated of a grey colour by fixed alkali, is decomposable by the nitric acid, and gives a superoxygenated muriat of mercury with muriatic acid. It is composed of

| | |
|---|---|
| Acid | 10 |
| Mercury | 76 |
| Oxyd | 11 |
| Water | 3 |
| | 100 |

Ammonia precipitates all these mercurial salts of a grey colour, but it forms at the same time a triple salt, namely, an ammoniaco-mercurial sulphat. It is formed in greater quantity with the mercurial sulphats with excess of acid, than with the neutral sulphat, and greater with this last than with the one with excess of oxyd. This salt is but little soluble in water; fixed alkali precipitates it white, ammonia dissolves it easily, and muriatic acid decomposes it. It contains

| | |
|---|---|
| Acid | 18 |
| Ammonia | 33 |
| Oxyd mercury | 39 |
| Water | 10 |
| | 100 |

It is the continuation of Cit. Fourcroy's labours on this subject, that he now communicates to the Institute; and our Correspondent thought it necessary to give the preceding very brief account of what has been already reported in the Tranasctions of the National Institute, in order to put our readers in a state to judge more easily of the the facts which are to be now described. Cit. Fourcroy observes, that in order to complete the labours on mercurial sulphats, we may prepare these different kinds of salts, not only by heating mercury with the sulphuric acid, but also by mixing this acid, or a soluble sulphat, with a nitric solution of mercury, more or less oxydated; we then obtain, according to circumstances, sulphats of different kinds. He then gives the proportions of acid, oxygen, and mercury which compose the different neutral sulphats, or acids, whether little or much oxydated. The nitrats of mercury have furnished Cit. Fourcroy with observations still more new and more important to sciences, than the sulphats. There are two kinds of nitrats; the one much, the other but little oxydated. The first is precipitated grey or almost black by the alkalis, and white by sulphats; it forms mild mercury with muriatic acid. The nitrat much oxydated, the consequence of strong and long continued ebullition, gives no precipitate with muriatic acid; it gives a yellow with sulphats, a white with ammonia, and an orange yellow with fixed alkalis. Nitric solutions of mercury, are often the mixture of two salts. That which is precipitated by water is the solution of oxyd much oxydated or red, in acid much concentrated. When we precipitate a nitric solution of mercury, but little oxydated by means of a fixed alkali, the first portion of the white somewhat coloured precipitate which is obtained is an insoluble and neutral nitrat of mercury, formed by an union of a portion of the separated oxyd with the remainder of the solution not decomposed. Citizen Fourcroy compares the properties of the nitrit and nitrat of mercury; almost all the solutions contain somewhat of this last salt. It is made by passing nitrous gas in the nitric solutions, which absorb it greedily; the superoxygenated nitrat is that which absorbs most. This last nitrat of mercury disengages much vapour by means of the sulphuric and nitric acids; it changes the skin to a deep purple, while the nitrat, much oxydated, turns it black; and the nitrat, but little oxydated, produces no change whatever. It keeps longer in the air than the alkaline nitrits, which absorb the oxygen, and pass into the state of nitrat. These nitrits are easily prepared by impregnating with nitrous gas the solutions of nitrats.

The Author means next to occupy himself with the muriats of mercury, of which he has discovered a new kind; as also with the sulphurs of this metal.

